# The Hemodynamic Management and Postoperative Outcomes After Cytoreductive Surgery and Hyperthermic Intraperitoneal Chemotherapy: A Prospective Observational Study

**DOI:** 10.1155/ccrp/8815211

**Published:** 2024-12-27

**Authors:** Sohan Lal Solanki, Vandana Agarwal, Reshma P. Ambulkar, Malini P. Joshi, Shreyas Chawathey, Shivacharan Patel Rudrappa, Manish Bhandare, Avanish P. Saklani

**Affiliations:** ^1^Department of Anesthesiology, Critical Care and Pain, Tata Memorial Hospital, Homi Bhabha National Institute, Mumbai, India; ^2^Department of Anesthesiology, Critical Care and Pain, Advanced Centre for Treatment Research and Education in Cancer, Tata Memorial Centre, Homi Bhabha National Institute, Navi Mumbai, India; ^3^Department of Anesthesia and Critical Care, West Suffolk NHS Foundation Trust, Bury St Edmunds, UK; ^4^Gastro-Intestinal and HPB Services, Department of Surgical Oncology, Tata Memorial Hospital, Homi Bhabha National Institute, Mumbai, India

**Keywords:** cytoreduction surgical procedures, fluid therapy, hemodynamic monitoring, hyperthermic intraperitoneal chemotherapy, postoperative complications

## Abstract

**Background:** Cytoreductive surgery with hyperthermic intraperitoneal chemotherapy (CRS-HIPEC) has become standard treatment for peritoneal cancers and metastases, significantly enhancing survival rates. This study evaluated the relationship between tumor burden, hemodynamic management, and postoperative outcomes after CRS-HIPEC.

**Methodology:** This study included 203 patients undergoing CRS-HIPEC. The study was registered with ClinicalTrials.gov (NCT02754115). Routine and advanced hemodynamic monitoring was performed. Data on fluid and blood transfusions, coagulation management, body temperature, blood gases, Peritoneal Carcinomatosis Index (PCI), and chemotherapeutic agents used were collected. Postoperatively, complications using the Clavien–Dindo classification were employed. Primary outcomes assessed PCI's impact on hemodynamic parameters and fluid management, with secondary outcomes including postoperative complications, mortality, and length of ICU and hospital stays.

**Results:** Patients with PCI > 20 experienced significantly longer surgeries (796.2 ± 158.3 min) as compared with patients with PCI 0–10 (551 ± 127 min) and patients with PCI between 11 and 20 (661.78 ± 137.7 min) (*p* ≤ 0.01). Patients with PCI > 20 received higher fluid requirements (mean: 5497.7 ± 2401.9 mL) as compared with PCI 0–10 (2631.2 ± 1459.9 mL) and PCI 10–20 (3964.65 ± 2044.6 mL) (*p* ≤ 0.01). Patients with PCI > 20 also had a prolonged ICU stays (median: 4 days) as compared with PCI 0–20 (median: 3 days). However, these differences were not significant in patients with PCI between 10 and 20. Significant differences in CI and SVI were observed among PCI groups during and after HIPEC. Significant differences were also observed among PCI groups for postoperative complications. Although 30-day survival rates varied clinically, they did not reach statistical significance.

**Conclusion:** A higher PCI score was significantly associated with increased duration of surgery, fluid requirements, the need for invasive hemodynamic monitoring, postoperative complications, and longer ICU stays. Tailoring perioperative strategies based on PCI scores has the potential to optimize these outcomes.

**Trial Registration:** ClinicalTrials.gov identifier: NCT02754115.

## 1. Introduction

Cytoreductive surgery with hyperthermic intraperitoneal chemotherapy (CRS-HIPEC) has become the standard of care for patients with primary peritoneal cancers and peritoneal metastasis from gastrointestinal and ovarian carcinomas [1, 2]. The procedure involves the removal of intraperitoneal tumor masses, which may include the excision of affected organs and, in some cases, stripping of the diaphragm. During the HIPEC phase, chemotherapeutic agents heated to temperatures between 41°C and 43°C are instilled into the abdominal cavity to enhance the cytotoxic effects of the chemotherapy [1, 2].

The Peritoneal Carcinomatosis Index (PCI) is a scoring system used to assess tumor burden in patients with peritoneal metastasis. It ranges from 0 to 39, based on the extent and distribution of the tumor in 13 abdominal regions, with each region assigned a score of 0 (no visible tumor), 1 (tumor less than 0.5 cm), 2 (tumor between 0.5 and 5 cm), and 3 (tumor larger than 5 cm) [2]. The PCI score is a crucial prognostic tool, as higher values correlate with more extensive disease and are associated with longer surgeries, increased blood loss, and significant fluid shifts. These factors contribute to significant hemodynamic instability, posing a challenge to perioperative management and leading to an elevated risk of postoperative morbidity and mortality.

CRS-HIPEC is known to cause considerable physiological stress. The combination of extensive organ resection and hyperthermic chemotherapy leads to fluid shifts, blood loss, and increased intra-abdominal pressure due to the infusion of 3-4 L of heated carrier solution. This rise in intra-abdominal pressure impairs venous return, reduces cardiac output, and can precipitate hemodynamic instability [2–6]. In addition, hyperthermia increases the risk of end-organ ischemia and renal dysfunction, especially in vulnerable patients. The procedure is associated with significant morbidity (20%–40%) and mortality (1%–5%), largely increased by these physiological challenges [2–4].

Intraoperative management during CRS-HIPEC requires careful attention to fluid balance and hemodynamic stability. Large volumes of fluid, often ranging from 8 to 12 mL/kg/h, may be necessary to maintain homeostasis due to the significant blood loss and fluid shifts. Hemodynamic monitoring plays a key role in guiding fluid therapy and the use of vasoactive agents to maintain cardiac output and adequate end-organ perfusion. Advanced hemodynamic parameters such as cardiac index (CI), Stroke Volume Index (SVI), and stroke volume variation (SVV) are essential for monitoring cardiovascular function and ensuring stable hemodynamics during surgery.

Given the complexity of CRS-HIPEC, the PCI score is a crucial factor influencing intraoperative hemodynamic management. Higher PCI scores often correlate with more severe intraoperative hemodynamic fluctuations, increased need for fluid resuscitation, and advanced hemodynamic monitoring to optimize patient outcomes. Despite its potential therapeutic benefits, the physiological stress induced by CRS-HIPEC necessitates careful perioperative management to reduce the risk of postoperative complications and mortality [7, 8].

This study aims to evaluate the relationship between tumor burden, perioperative hemodynamic management, fluid therapy, and postoperative outcomes in patients undergoing CRS-HIPEC. We hypothesize that effective hemodynamic management during and after the procedure is associated with improved outcomes, including a reduction in complications, shorter hospital stays, and lower postoperative morbidity and mortality.

## 2. Methodology

This prospective observational study was conducted from April 2016 to December 2022 after approval from Institutional Ethics Committee (IEC). The inclusion criteria for the study were primary peritoneal cancers, gastrointestinal and gynecological cancer patients posted for CRS-HIPEC, aged between 18 and 70 years, and belonging to the American Society of Anesthesiologists physical status class I–III. Waiver of consent was granted by IEC in view of the observational nature of the study. Patients in whom HIPEC was canceled after CRS because of change in surgical plan were excluded from the study. The study was conducted as per principles of the Declaration of Helsinki (2013) and its subsequent amendments.

In the preoperative period, patients' disease status, medical history, preanesthetic evaluation, laboratory investigations, radiological imaging, and other relevant clinical data were reviewed and recorded.

In the operating room, standard monitoring included continuous measurement of heart rate, blood pressure, oxygen saturation, and temperature, with documentation in the anesthesia chart. Anesthesia induction was performed using intravenous propofol and fentanyl, along with muscle relaxants such as atracurium or vecuronium, as appropriate. An arterial catheter was placed in all patients for invasive blood pressure monitoring and advanced hemodynamic monitoring. Central venous catheter placement was also performed for administering vasopressors if needed.

Hemodynamic monitoring was enhanced using the FloTrac system with the EV1000 monitoring platform (Edwards Lifesciences) connected to the arterial line. Parameters such as CI, cardiac output (CO), stroke volume (SV), SVI, and SVV were recorded and utilized to guide goal-directed fluid therapy (GDFT). The objective of fluid management during the perioperative period was to maintain CI, SVV, SVI, and pulse pressure variation (PPV) within 20% of the baseline values, established after connecting the FloTrac system.

The main objective of anesthesia management during CRS-HIPEC included a balanced anesthetic technique to ensure optimal hemodynamic stability, oxygenation, and normothermia. The FloTrac system with the EV1000 platform was used as a key tool for hemodynamic monitoring, providing real-time measurements of dynamic parameters such as CI and SVI. This allowed precise adjustments in fluid administration and vasopressors. The anesthesia team adhered to GDFT protocols, which emphasized maintaining parameters within a 20% range to reduce intraoperative hypotension and maintain organ perfusion. The use of low dose norepinephrine (often referred to as “baby norepinephrine”), was adjusted as needed to support hemodynamic goals.

Data on patient characteristics, anesthetic management, intraoperative fluid and blood transfusion, coagulation management, body temperature, and delta temperature (defined as the difference between maximum and minimum temperatures measured throughout the CRS-HIPEC procedure), as well as arterial and venous blood gas measurements, were collected. At the conclusion of the procedure, the PCI score, the number of organs resected, the number of anastomoses performed, diaphragmatic stripping (if performed), the completeness of cytoreduction (CC) score—where CC-0 indicates no visible residual tumor, CC-1 indicates residual tumors up to 2.5 mm, CC-2 indicates residual tumors between 2.5 mm and 2.5 cm, and CC-3 indicates residual tumors larger than 2.5 cm, and the chemotherapeutic agent(s) administered during HIPEC were documented. The duration of each phase of the surgery, along with the total surgical time, was also recorded. The HIPEC protocol involves the use of mitomycin-C (15 mg/m^2^ of body surface area [BSA]) and doxorubicin (15 mg/m^2^ of BSA) for pseudomyxoma peritonei, mesothelioma, colorectal, and appendiceal cancers. For gastric and ovarian cancers, cisplatin (75 mg/m^2^ of BSA) is used. HIPEC is performed for 90 min using the open “Coliseum” technique, with peritoneal dialysis solution (3 L) serving as the carrier solution. Sodium thiosulfate was not used in any of the patients due to its unavailability.

Temperature control during CRS was managed using fluid warmers and warming blankets to maintain normothermia. During the HIPEC phase, warming devices were discontinued, and cold fluids along with cool air blankets were used to keep the core body temperature below 39°C. In the postoperative period, if a patient developed hypothermia, fluid warmers and warming devices were used as needed to restore and maintain normothermia.

The postoperative course, including complications classified according to the Clavien–Dindo (CD) classification [9] (Table 1), was tracked. The postoperative intensive care unit (ICU) stay and morbidity were assessed using the Sequential Organ Failure Assessment (SOFA) score [10] (Table 2). In addition, the length of stay in both the ICU and hospital, as well as 30- and 90-day mortality, were recorded.

We classified patients based on their PCI score into three groups: PCI < 10, PCI 11–20, and PCI > 20, representing the extent of disease. This classification was used to assess intraoperative hemodynamics, fluid therapy, and perioperative outcomes. The primary outcome of this study was to evaluate the impact of PCI scores on hemodynamic monitoring parameters, including CI, CO, SVI, delta SVI, PPV, SVV, and fluid management. Secondary outcomes included postoperative complications according to the CD classification, 30- and 90-day mortality, and the length of ICU and hospital stay.

### 2.1. Statistical Analysis

Demographic, clinical, and disease-related variables were presented as frequencies (percentages), means (standard deviations), or medians (interquartile ranges), as appropriate. Parametric or nonparametric statistical tests were used based on the distribution of the data (normally distributed or not). The Kolmogorov–Smirnov test was used to assess the normality of baseline characteristics among patients classified into different PCI groups. For non-normally distributed data, the Mann–Whitney *U* test and Kruskal–Wallis test were employed, while ANOVA was applied for normally distributed data to evaluate associations among various perioperative parameters and related outcomes. Fisher's exact test was used to compare expected and observed outcomes and to assess associations between categorical variables. Univariable and multivariable logistic regression analyses were performed to identify significant associations between perioperative parameters and postoperative outcomes. A *p* value of less than 0.05 was considered statistically significant.

## 3. Results

A total of 203 patients underwent CRS-HIPEC during the study period. The age, gender, primary malignancies, and duration of surgery for different PCI groups are detailed in Table 1. Information on chemotherapeutic drugs used, total fluid administered, blood transfusions, CC score, number of organs resected, number of anastomoses, delta-temperature, complications, and duration of hospital stay are provided in Table 2.

Patients with a greater burden of disease who underwent extensive resections (PCI > 20) had a significantly longer mean duration of surgery (796.2 ± 158.3 min) compared with those with a PCI between 0 and 10 (551 ± 127 min) and a PCI between 11 and 20 (661.78 ± 137.7 min) (*p* ≤ 0.01) (Table 3).

The mean volume of fluids administered, excluding blood and blood products, in different PCI groups is detailed in Table 3. The volume of intraoperative fluids administered was found to be statistically significant across all three PCI score-based patient groups (Pearson's Chi-square: 48.315; *p* ≤ 0.01) (Table 3). The correlation between IV fluid given during CRS and various perioperative parameters are mentioned in Appendix table 2.

Baseline mean CI values were significantly different among patients with PCI scores of 0–10 (3.4931), 11–20 (2.8043), and > 20 (3.3700) (ANOVA: sum of squares: 79.028; *p* ≤ 0.01).

**Figure 1:**
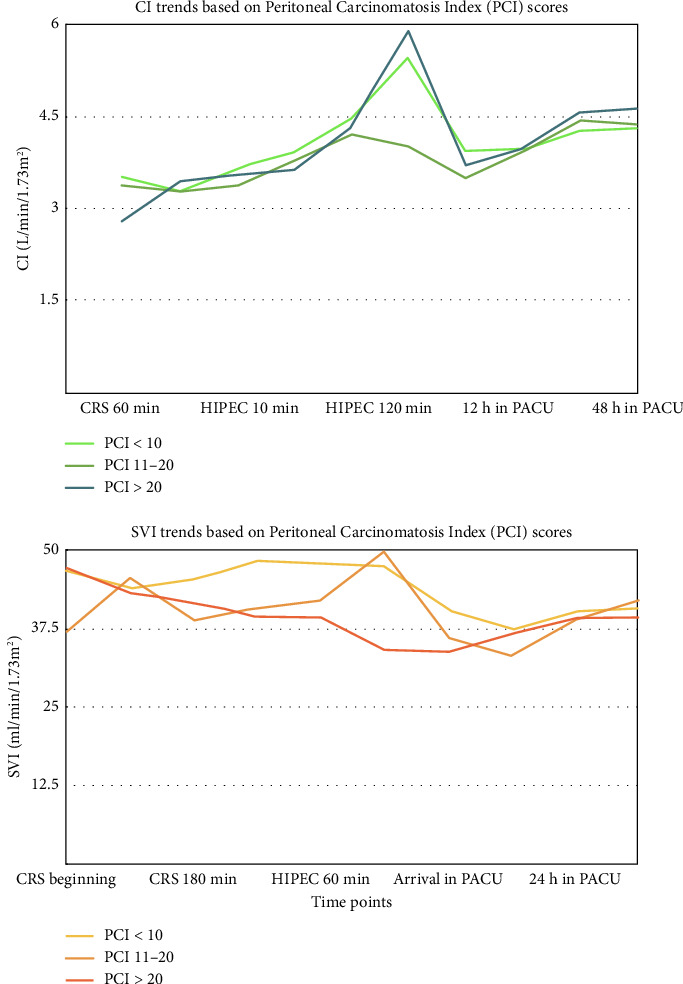
Intraoperative and postoperative trends in mean cardiac index (CI) (left panel) and Stroke Volume Index (SVI) (right panel) in patients, classified based on the Peritoneal Carcinomatosis Index (PCI) score. CI in patients with a PCI score > 20 was lower at all points in time compared with those with PCI up to 20. The CI in patients with PCI score up to 20 peaked at around 120 min of HIPEC and lowered, to a value higher than the baseline CI at the start of CRS. Similarly, SVV in patients with a PCI score > 20 was lower than patients belonging to the less high PCI scores throughout the perioperative period from the start of CRS till 24 h after the surgical procedure is complete.

The mean SVI values were significantly different at 10 and 60 min after starting HIPEC in patients with PCI scores of 0–10 (SVI-10 min: 48.29; SVI-60 min: 47.97), 11–20 (SVI-10 min: 41; SVI-60 min: 42.11), and > 20 (SVI-10 min: 39.5; SVI-60 min: 39.48) (ANOVA: SVI CRS beginning: sum of squares: 16,493.679, *p*=0.002; HIPEC 10 min: sum of squares: 28,393.892, *p* ≤ 0.01; SVI HIPEC 60 min: Sum of squares: 25,613.411, *p* ≤ 0.01) (Table 3 and Figure 1).

Baseline mean CI values were significantly different among patients with PCI scores of 0–10 (3.4931), 11–20 (2.8043), and > 20 (3.3700) (ANOVA: sum of squares: 79.028; *p* ≤ 0.01). However, these differences were not observed in mean CI values during the intraoperative or postoperative periods (Table 3 and Figure 1).

**Figure 2:**
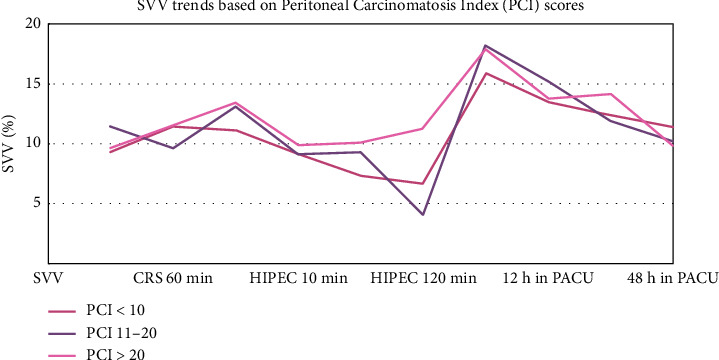
Intraoperative and postoperative trends in Stroke volume variation based on PCI score. The values of SVV show a peak at two points in the perioperative period in patients belonging to all PCI classes, which occur at the end of CRS and before beginning of HIPEC and another at the time of PACU admission. Patients with PCI score < 10 had the highest SVV throughout the perioperative period compared with the corresponding SVV values in patients with PCI 11–20 and > 20. Conversely, patients with PCI > 20 had lowest SVV throughout the perioperative period. PACU, postanesthesia care unit.

Mean SVV values were significantly different 60 min after starting HIPEC in patients with PCI scores of 0–10 (7.3656), 11–20 (9.2941), and > 20 (10.1250) (ANOVA: SVV at HIPEC 60 min: sum of squares: 4344.659; *p* ≤ 0.01). However, there were no statistically significant differences in SVV trends during the CRS or postoperative periods (Table 3 and Figure 2).

We found that the mean body temperature did not significantly change during CRS from the initial temperature. However, body temperature decreased by about 0.6°C–0.8°C 10 min after commencing HIPEC, followed by an increase of 1.3°C–1.4°C during HIPEC, compared with the beginning of CRS. There was no significant difference in mean temperature at any corresponding time point based on PCI scores during the perioperative period (Appendix Table 1). The mean temperature after patients' arrival to the ICU was 35.6°C (SD: 1.91). Of the 203 patients we studied, 145 patients had a recorded temperature on shifting to the ICU. Of these, 44 patients (30.3%) were hypothermic (< 35°C core body temperature) on arrival to the ICU.

Primary diagnosis (disease), the number of organs resected during CRS, and the number of anastomoses done were analyzed for their impact on postoperative complications. Primary diseases significantly affected postoperative complications (*p* ≤ 0.01), while the number of organs resected (*p*=0.258) and anastomoses done (0.431) did not affect the postoperative complications (Table 4).

Interestingly, diaphragmatic stripping did not independently increase ICU stay length (Mann–Whitney U: 1488.5; *p*=0.602) or in the regression model. However, diaphragmatic stripping was significantly associated with the need for mechanical ventilation for 24 h or more (Mann–Whitney U: 2709.5; *p* ≤ 0.01). Total blood loss, need for mechanical ventilation for 24 h or more, duration of surgery, CC score after CRS, blood loss during CRS, total number of PRBCs transfused during the procedure, and PCI score were highly significant on univariable logistic regression.

Renal function was assessed using serum creatinine values. The mean (SD) of creatinine levels on postoperative days (PODs) 1, 3, and 5 were recorded (Table 3). On POD 1, two patients had serum creatinine levels greater than 1.5 mg/dL (2.06 and 1.60 mg/dL), and in both patients, serum creatinine levels normalized by POD 3. On POD 3, one patient had a serum creatinine level of 1.80 mg/dL, which increased to 3.00 mg/dL by POD 5, and this patient required renal replacement therapy. Another patient had a serum creatinine level of 1.5 mg/dL on POD 3, which normalized by POD 5. These differences between the various PCI groups were statistically significant on POD 3 (*p* ≤ 0.01).

The median duration of ICU stay varied across patients with different PCI scores. Patients with PCI > 20 had a median ICU stay of 4 days (IQR: 3–7 days), compared with 3 days (IQR: 2–3 days) for those with PCI scores between 0–10 and 3 days (IQR: 2–4 days) for those with PCI scores between 10 and 20. (Kruskal–Wallis *H* = 22.495; *p* ≤ 0.01).

The 30-day survival rates were 99.1% for patients with PCI 0–10, 95.5% for those with PCI 11%–20%, and 93.3% for those with PCI > 20. While this difference is clinically significant, it was not statistically significant (Pearson's Chi-square: 2.354; *p*=0.308).

The 30-day readmission rates were comparable across all PCI groups (Pearson's Chi-square: 0.849; *p*=0.654) (Table 3).

We analyzed the effect of these seven parameters viz., total blood loss, need for mechanical ventilation for 24 h or more, duration of surgery, CC score, blood loss during CRS, total number of PRBCs transfused during the procedure, and PCI score on ICU stay length using multivariate logistic regression and found that mechanical ventilation for 24 h or more and CC score at the end of CRS predicted an ICU stay longer than 8 days (reference outcome: ICU stay of up to 7 days) (Table 5).

On univariable logistic regression, SVI at 60 min after starting HIPEC, ICU stay longer than 7 days, and readmission within 30 days of surgery significantly predicted 30-day survival. However, ICU stay longer than 7 days and maximum SVV during HIPEC phase were predictive of 30-day survival on multivariable logistic regression (Table 6).

On multivariable analysis, average heart rate and average CO during CRS and end-tidal carbon dioxide during reconstructive phase were predictive of postoperative complications after CRS-HIPEC (Table 7).

A total of 21 out of 203 patients (10.34%) were readmitted within 30 days after surgery. The reasons for readmission included sepsis (3/21; 14.28%), electrolyte imbalances such as hyperkalemia (3/21; 14.28%), medical conditions (e.g., dengue fever and acute exacerbation of asthma) (3/21; 14.28%) patients, intestinal obstruction (2/21; 9.52%), nutritional support (1/21; 4.76%), further management of residual disease (2/21; 9.52%), perforation peritonitis (1/21; 4.76%), anastomotic leaks (2/21; 9.52%), and short bowel syndrome (1/21; 4.76%). In 3 patients, the reason for readmission was not found on record (14.28%).

Of the 203 patients operated, 7 patients died in hospital. One patient had cardiac arrest after hemodialysis for hyperkalemia after undergoing re-exploration surgery for anastomotic leak. Six patients had severe sepsis and septic shock with multi organ dysfunction syndrome and died.

## 4. Discussion

This study focuses on highlighting major perioperative concerns in CRS-HIPEC, including duration of surgery, blood loss, delta temperature, PCI, and fluid management. To the best of our knowledge, this is one of the largest cohorts studied for hemodynamic parameters in CRS-HIPEC.

Large fluid shifts are common during cytoreduction, and intraoperative fluid losses can reach up to 8–12 mL/kg and require adequate crystalloids and colloids to ensure adequate perfusion pressure and urine output without causing fluid overload [2]. Patients with lower cardiopulmonary reserve may not tolerate high volumes of intravenous fluids and may need vasopressors/inotropes based on the extent of their ability to tolerate fluids, as also their fluid responsiveness. Liberal fluid strategy, with fluid as high as 1500 mL/h given to patients described in some studies, has been shown to cause several complications, including fluid overload, surgical site edema, end organ dysfunction, and greater length of ICU and hospital stay [11–15].

Conversely, restrictive fluid strategies may compromise end-organ perfusion, particularly during the hemodynamic fluctuations during CRS-HIPEC, leading to renal dysfunction reported in few studies [13, 16, 17]. However, a retrospective study has showed that restrictive fluid strategy decreased the length of hospital stay (11.5 vs. 9.7 days; *p* ≤ 0.01) and 60-day postoperative complications (28% vs. 45%; *p*=0.02), with incidence of postoperative renal dysfunction and the highest serum creatinine levels not significantly different in the restrictive versus permissive fluid therapy groups [13]. CRS-HIPEC procedure is known to cause acute kidney injury (AKI) in the postoperative period, and apart from the decreased renal perfusion, use of cisplatin chemotherapy alone or in combination is an important cause of AKI [18].

GDFT approach which involve fluid administration based on CI, SVI, or SVV is well-suited for CRS-HIPEC, where physiological challenges of major surgery and fluid shifts, and hyperthermic environment leads to difficulty in maintaining hemodynamic stability and adequate tissue oxygenation [19, 20].

In our study, fluid administration in patients with three different PCI groups showed a highly significant difference (*p* ≤ 0.01), suggesting that patients with higher PCI scores need aggressive fluid management. In addition, significant variations were observed in the mean CI and mean SVI at various stages of the CRS-HIPEC, particularly at the beginning of CRS and during HIPEC, which indicates the impact of PCI on cardiac function. Mean SVV at 60 min of HIPEC phase also showed significant difference (*p* ≤ 0.01), highlighting the hemodynamic instability in patients with higher PCI scores. The use of FloTrac system with the EV1000 platform ensured optimal hemodynamic stability and organ perfusion during the procedure. While some groups focus on the duration of mean arterial pressure (MAP) below 60 mmHg, our protocol prioritized maintaining hemodynamic parameters within the defined range to minimize hypotensive episodes. Emerging technologies, such as machine learning-based early warning systems (e.g., HYPE Randomized Clinical Trial) [21] and predictive models for intraoperative hypotension (e.g., Hypotension Prediction Index (HPI)) [22] were shown to have good clinical outcomes, but their application in our study was not implemented except for 4 cases where we used HPI in addition to the FloTrac [23].

As per the international expert consensus, the PCI score is crucial in decision-making for CRS-HIPEC. In patients with colorectal cancer, a PCI score ≤ 16 is acceptable for CRS-HIPEC, whereas a PCI scores > 20 may indicate a need for alternative treatments and supportive care [24]. However, optimal PCI score cutoff is not yet determined for some cancers like gastric cancer and pseudomyxoma peritonei [25, 26].

Esteve-Perez et al. [14] studied 92 adult patients with colorectal and ovarian malignancies undergoing CRS-HIPEC and found significant interindividual variation in the fluid requirement. They also observed that PCI and duration of surgery, fluid therapy, and intraoperative transfusion percentage were highly correlated (*p* < 0.02). Similar to these findings, we found that all three of the PCI score-based patient groups showed a statistically significant variation in the volume of intraoperative fluids given. This was found to be clinically relevant, as larger burden of disease necessitates more extensive and technically complex surgical resection, entailing greater blood loss and thus need for more fluids to be administered during surgery. Higher PCI values are a major predictor of postoperative morbidity, especially for scores > 20.

High volume diseases (greater PCI) lead to increased blood loss and higher fluid requirements during CRS-HIPEC [14, 15, 27, 28]. Multivariable analyses from previous studies underscore age, intraoperative fluid rates, and blood loss as independent predictors of postoperative morbidity in CRS-HIPEC [11, 15]. Specifically, higher fluid infusion rates (> 15.7 mL/kg/h) have been associated with increased comprehensive complication indices and prolonged ICU stays [11].

On multivariable analysis, we found that higher PCI scores were associated with prolonged mechanical ventilation (> 24 h), increased 30-day hospital readmission rates, and reduced 30-day survival. We also found a positive correlation between total fluid administered and ICU stay, but there was no significant association between total fluid administered and 30-day survival, suggesting that other factors may influence survival outcomes.

The identification of predictors for prolonged ICU stays and 30-day survival enhances risk stratification and prognostication in CRS-HIPEC. The significance of mechanical ventilation for 24 h or more and the CC score underscores their utility as key determinants of postoperative recovery and overall survival, as found in our study. These findings are similar to what were found by Macrì et al. in their retrospective analysis of outcomes of CRS-HIPEC in patients with ovarian malignancy [27].

In this study, 30.3% patients had a core body temperature < 35°C on arrival to ICU (rebound hypothermia). The hypothermia in postoperative period may be because of failure to restart the warming devices after completion of HIPEC phase and because of the higher delta temperature.

The emphasis of our study on tailoring patient care based on disease extent (PCI scores) and identified perioperative predictors allows for a more individualized approach to the perioperative care of patients undergoing CRS-HIPEC. Our findings align with the existing literature on the challenges associated with the perioperative management of CRS-HIPEC. The prolonged duration of surgery in patients with extensive disease corroborates studies highlighting the intricacies of extensive resections and their subsequent impact on procedural timelines. The pattern of hemodynamic parameters observed in this study underscores their critical role in the perioperative management of CRS-HIPEC patients.

Our study has certain limitations. First, as a single-center study, the generalizability of the findings to different geographical and healthcare settings is limited. Second, variations in clinical practices, surgical techniques, and postoperative care protocols may lead to differences in outcomes. In addition, our study lacked a predefined sample size, as we enrolled consecutive CRS-HIPEC patients.

## 5. Conclusion

Our study highlights significant variations in CI, SVI, and fluid requirements across different PCI groups. A higher PCI score is significantly associated with increased duration of surgery, fluid requirements, the necessity for invasive hemodynamic monitoring, postoperative complications, and ICU stay. Tailoring perioperative strategies based on PCI scores has the potential to optimize these outcomes.

## Figures and Tables

**Table 1:** Baseline characteristics.

Characteristics	Value
Age, years, median (IQR)	49 (37–58)
Sex, *n* (%)	
Male	89 (43.8)
Female	114 (56.2)
BMI (kg/m^2^), median (IQR)	22.6 (20.5–25.2)
Diagnosis, *n* (%)	
Colorectal cancer	69 (34.0)
Stomach cancer	35 (17.2)
Ovarian cancer	34 (16.7)
Pseudomyxoma peritonei	30 (14.8)
Carcinoma of the appendix	24 (11.8)
Mesothelioma	8 (3.9)
Others	3 (1.5)
ASA class, *n* (%)	
1	119 (58.6)
2	77 (37.9)
3	7 (3.4)
PCI, median (IQR), [range]	10 (5–18), [0–39]
0–10	113 (55.66)
11–20	45 (22.16)
> 20	45 (22.16)
Duration of surgery (min), median (IQR)	602.5 (510–730)

Abbreviations: ASA, American Society of Anesthesiologists; BMI, Body Mass Index; IQR, interquartile range; PCI, Peritoneal Carcinomatosis Index.

**Table 2:** Intraoperative and postoperative details.

Intraoperative and postoperative details	Values
Chemotherapeutic agents instilled, *n* (%)	
Doxorubicin + mitomycin	106 (52.2)
Cisplatin + mitomycin	50 (24.6)
Doxorubicin + cisplatin	25 (12.3)
Oxaliplatin	10 (4.9)
Others	12 (5.9)
Fluid received during surgery (mL), mean (SD; range)	
During CRS	3557.8 (2183.94; 0–11,500)
During HIPEC	2890.96 (1965.34; 250–9000)
Intraoperative transfusions, mL, mean (SD; range)	513.96 (563.11, 0-3252)
Blood loss, mL, mean (SD; range)	
During CRS	2352.21 (1912.52; 100–12,000)
During HIPEC	280.31 (337.88; 100–3000)
During reconstruction	121.35 (299.08; 0–2700)
Total blood loss	2619.73 (1997.38; 200–13,000)
CC score, *n* (%)	
0	174 (87.5)
1	24 (11.8)
2	5 (2.5)
Organs resected	
1	46 (22.7%)
2	50 (24.6%)
> 2	107 (52.7%)
Number of anastomosis	
0	10 (4.9%)
1	127 (62.6%)
> 1	66 (32.5%)
Delta temperature, degree celsius, mean (SD; range)	2.12 (1.106; 1.4–5.4)
Rebound hypothermia (temperature < 35°C on arrival in ICU), *N* = 145	44 out of 145 (30.3%)
Length of hospital stay, number of days, median (IQR)	10 (9–14)
Mortality, *n* (%)	7 (3.45)
Complications (Clavien–Dindo classification), *n* (%)	
No complications (CD 0)	32 (15.76)
Minor complications (CD 1-2)	139 (68.47)
Major complications (CD 3-4)	25 (12.31)
Postoperative in-hospital death (CD 5)	7 (3.45)
30-day survival, *n* (%)	196 (96.5)
90-day survival, *n* (%)	193 (95.1)

*Note:* CD, Clavien–Dindo class.

Abbreviations: CC score, cytoreduction completion score; CRS, cytoreductive surgery; HIPEC, hyperthermic intraperitoneal chemotherapy; IQR, interquartile range; SD, standard deviation.

**Table 3:** Intraoperative and postoperative characteristics and their relationship with postoperative considerations.

Characteristic	All patients	PCI score groups			Test of significance		Significance (*p* value)
		0–10	11–20	> 20	Value	df	
Duration of surgery (min, mean (SD))	630.27 (168.65)	551 (127)	661.78 (137.7)	796.2 (158.3)	ANOVA sum of square: 1,988,536.02	53.06	≤ 0.01

Fluid given during surgery, N = 195, mean (SD)							
Mean amount of fluid given (mL) (SD)	3557.8 (2183.94)	2631.2 (1459.9)	3964.65 (2044.6)	5497.7 (2401.9)	Pearson's chi-square: 48.315	2	≤ 0.01

**Mean cardiac index (CI) (L/min/** **m** ^2^ **)**							
					**ANOVA sum of squares:**	**df**	

At the beginning of CRS	3.33	3.49	2.80	3.37	79.028	116	≤ 0.01

**Mean Stroke Volume Index (SVI) (mL/** **m** ^2^ **)**							
					**ANOVA sum of squares:**	**df**	

CRS beginning	44.94	46.72	37.14	47.2	16,493.679	111	≤ 0.01
HIPEC 10 min	44.76	48.29	41.00	39.50	28,393.892	166	≤ 0.01
HIPEC 60 min	44.68	47.96	42.11	39.48	25,613.411	162	≤ 0.01

**Mean Stroke Volume Variation (SVV), %**							
					**ANOVA sum of squares:**	**df**	

HIPEC 60 min	8.42	7.36	9.29	10.12	4344.659	166	≤ 0.01

**Serum creatinine, mg/dL, mean (SD)**							
					**ANOVA sum of squares:**	**df**	

POD 1	0.66 (0.24)	0.65 (0.24)	0.71 (0.26)	0.63 (0.21)	10.61	177	0.34
POD 3	0.62 (0.23)	0.54 (0.17)	0.76 (0.32)	0.62 (0.19)	7.13	130	≤ 0.01
POD 5	0.59 (0.34)	0.55 (0.20)	0.69 (0.57)	0.57 (0.26)	10.30	87	0.29

**Clavien**–**Dindo class**					**Pearson**'**s chi-square:**	**df**	**Significance (** **p** **value)**

Class 0	32	25 (22.1)	5 (11.4)	2 (4.5)	25.857	10	≤ 0.01
Class 1	49	30 (26.5)	12 (27.3)	7 (15.6)			
Class 2	90	51 (45.1)	19 (43.2)	20 (44.4)			
Class 3	18	4 (3.5)	5 (11.4)	9 (20)			
Class 4	7	2 (1.8)	1 (2.3)	4 (8.9)			
Class 5 (death)	7	2 (1.8)	2 (4.5)	3 (6.7)			

*Postoperative outcomes*							
No of patients who survived 30 days after surgery *N* (%) (*N* = 202)	196 (96.5)	111 (54.9)	43 (21.3)	42 (20.8)	Pearson's Chi-square: 2.354	2	0.308
ICU discharge day (POD), median (IQR)	3 (2–4)	3 (2-3)	3 (2–4)	4 (3–7)	Kruskal–Wallis: *H* = 22.495	2	≤ 0.01
No. of patients readmitted within 30 days after discharge (%*N*) (*N* = 202)	21 (10.39)	13 (6.4)	5 (2.48)	3 (1.48)	Pearson's Chi-square: 0.849	2	0.654

*Note:* CD, Clavien–Dindo class.

Abbreviations: CC score, cytoreduction completion score; CRS, cytoreductive surgery; HIPEC, hyperthermic intraperitoneal chemotherapy; IQR, interquartile range; POD, postoperative day; SD, standard deviation.

**Table 4:** Primary diseases, surgical factors, and postoperative complications.

Characteristic		Postoperative complications (Clavien–Dindo classification)			Test of significance		(*p* value)
		None CD 0	Minor CD 1-2	Major CD 3–5	Value	df	
Primary Disease, *n*	Ca appendix	0	32	1	54.804	12	≤ 0.01
	Ca Colo-rectal	5	51	13			
	Ca ovary	10	23	1			
	Ca stomach	12	22	1			
	Mesothelioma	2	3	3			
	PMP	2	16	12			
	Others	0	1	2			

Number of organs resected, *n*	1	8	33	5	5.295	4	0.258
	2	4	39	7			
	> 2	19	67	21			

Number of anastomosis, *n*	0	2	8	0	3.816	4	0.431
	1	22	85	20			
	> 1	7	46	13			

*Note:* CD, Clavien–Dindo class.

Abbreviation: PMP, Pseudomyxoma Peritonei.

**Table 5:** Multivariable analysis of intraoperative and postoperative characteristics and their relationship with need for prolonged ICU stay for more than 7 days.

Independent variable	OR_adjusted_ (CI 95%)	*p* value
Baseline albumin value	0.341 (0.129–0.901)	0.03
Length of surgery (min)	1.003 (0.998–1.007)	0.788
PCI	0.934 (0.850–1.025)	0.308
CC score	4.760 (1.118–20.272)	0.035
Blood loss during CRS	1.000 (0.998–1.002)	0.843
Total blood loss	0.998 (1.000–1.002)	0.922
PRBC transfusions	1.000 (0.998–1.001)	0.638
POD of extubation	1.156 (0.814–1.641)	0.418
Need for mechanical ventilation for > 24 h	0.233 (0.055–0.998)	0.05

Abbreviations: BMI, Body Mass Index; CC score, cytoreduction completion score; CRS, cytoreductive surgery; HIPEC, hyperthermic intraperitoneal chemotherapy; IQR, interquartile range; POD, postoperative day; PRBC, packed red blood cells; SD, standard deviation; SVI, Stroke Volume Index.

**Table 6:** Multivariate analysis of intraoperative and postoperative characteristics and their relationship with postoperative 30-day survival.

Independent variable	OR_adjusted_ (CI 95%)	*p* value
SVI at 60 min of HIPEC	0.862 (0.742–1.003)	0.862
Maximum SVV during CRS	1.125 (1.024–1.235)	0.013
ICU stay for more than 7 days	14.115 (1.028–193.861)	0.048
Readmission in 30 days of surgery	0.115 (0.005–2.902)	0.189

Abbreviations: BMI, Body Mass Index; CI, confidence interval; CRS, cytoreductive surgery; HIPEC, hyperthermic intraperitoneal chemotherapy; IQR, interquartile range; SD, standard deviation; SVI, Stroke Volume Index.

**Table 7:** Multivariable analysis of intraoperative and postoperative characteristics and their relationship with postoperative complications.

Independent variable	OR_adjusted_ (CI 95%)	*p* value
CRS average heart rate	1.07 (1.03–1.112)	≤ 0.01
CRS average cardiac output	0.587 (0.377–0.912)	0.018
End-tidal carbon dioxide during reconstructive phase	0.808 (0.696–0.938)	≤ 0.01

## Data Availability

The data supporting the findings of this study are available from the corresponding author upon reasonable request. Access to the data may be subject to certain restrictions due to privacy considerations, legal obligations, or ethical requirements. These restrictions are in place to ensure compliance with relevant data protection regulations and to uphold the confidentiality and integrity of participant information. Requests for access will be evaluated on a case-by-case basis and may require approval from institutional ethics committee.

## Supporting Information

Appendix Table 1: Intraoperative temperature trends based on the PCI score.

Appendix Table 2: Spearman's rho test to correlate associations between various perioperative factors and volume of fluid given during CRS.
