# Supplementary material for: The Hemodynamic Management and Postoperative Outcomes After Cytoreductive Surgery and Hyperthermic Intraperitoneal Chemotherapy: A Prospective Observational Study
Source: Crit Care Res Pract. 2024 Dec 27;2024:8815211. doi: 10.1155/ccrp/8815211 (PMC11698608; doi:10.1155/ccrp/8815211)
Supplement: Supporting Information — Additional supporting information can be found online in the Supporting Information section. [file 8815211.f1.docx]

**The hemodynamic management and postoperative outcomes after cytoreductive surgery and hyperthermic intraperitoneal chemotherapy: A prospective observational study**

**APPENDIX (SUPPLEMENTARY TABLES)**

**Appendix Table 1**: Intraoperative temperature trends based on PCI score

| Mean Temperature (deg C) | PCI Score | | | ANOVA | | |
| --- | --- | --- | --- | --- | --- | --- |
|  | 0-10 | 11-20 | >20 | Sum of squares | df | p-value |
| At the beginning of CRS | 35.6058 | 35.3400 | 35.5902 | 94.558 | 184 | 0.123 |
| At CRS 60 min | 35.5869 | 35.3857 | 35.6558 | 73.007 | 191 | 0.101 |
| At CRS 180 min | 35.6260 | 35.3375 | 35.6605 | 94.997 | 178 | 0.071 |
| At HIPEC 10 min | 34.9304 | 34.5837 | 34.6465 | 182.741 | 197 | 0.070 |
| At HIPEC 60 min | 36.9358 | 36.6738 | 36.9951 | 154.800 | 186 | 0.184 |
| At HIPEC 120 min | 36.5000 | 35.8000 | 37.0250 | 23.125 | 10 | 0.572 |

*ANOVA, Analysis of Variance; CRS, Cytoreductive Surgery; HIPEC, Hyperthermic Intraperitoneal Chemotherapy; PCI, Peritoneal Carcinomatosis Index*

**Appendix Table 2**: Spearman’s rho test to correlate associations between various perioperative factors and volume of fluid given during CRS

| **Correlation between IV fluid given during CRS and various perioperative parameters** | | | |
| --- | --- | --- | --- |
| **Variable** | **Spearman’s rho** | **p-value** | **N** |
| 30 day survival | 0.136 | 0.059 | 160 |
| Readmission in 30 days | -0.078 | 0.277 | 160 |
| **Clavien-Dindo class** | **0.301** | **≤ 0.01** | **160** |
| **90-day survival** | **0.243** | **≤ 0.01** | **160** |
| ScVO2 CRS Start | -0.053 | 0.509 | 160 |
| ScVO2 CRS end | -0.543 | 0.266 | 6 |
| **ICU stay** | **0.220** | **≤ 0.01** | **160** |

*CRS, Cytoreductive Surgery; ICU, Intensive Care Unit; ScVO2, Central Venous Oxygen Saturation.*
